# Tumor Mutation Burden-Associated LINC00638/miR-4732-3p/ULBP1 Axis Promotes Immune Escape *via* PD-L1 in Hepatocellular Carcinoma

**DOI:** 10.3389/fonc.2021.729340

**Published:** 2021-09-08

**Authors:** Feng Qi, Xiaojing Du, Zhiying Zhao, Ding Zhang, Mengli Huang, Yuezong Bai, Biwei Yang, Wenxing Qin, Jinglin Xia

**Affiliations:** ^1^Liver Cancer Institute, Zhongshan Hospital, Fudan University, Shanghai, China; ^2^Department of Oncology, Second Affiliated Hospital of Naval Medical University, Shanghai, China; ^3^Minhang Branch, Zhongshan Hospital, Fudan University, Shanghai, China; ^4^The First Affiliated Hospital of Wenzhou Medical University, Wenzhou, China; ^5^The Medical Department, 3D Medicines Inc., Shanghai, China

**Keywords:** hepatocellular carcinoma, LINC00638, ULBP1, tumor mutation burden, PD-L1

## Abstract

Tumor mutation burden (TMB) is associated with immune infiltration, while its underlying mechanism in hepatocellular carcinoma (HCC) remains unclear. A long noncoding RNA (lncRNA)-related competitive endogenous RNA (ceRNA) network can regulate various tumor behaviors, and research about its correlation with TMB and immune infiltration is warranted. Data were downloaded from TCGA and ArrayExpress databases. Cox analysis and machine learning algorithms were employed to establish a lncRNA-based prognostic model for HCC. We then developed a nomogram model to predict overall survival and odds of death for HCC patients. The association of this prognostic model with TMB and immune infiltration was also analyzed. In addition, a ceRNA network was constructed by using DIANA-LncBasev2 and the starBase database and verified by luciferase reporter and colocalization analysis. Multiplex immunofluorescence was applied to determine the correlation between ULBP1 and PD-L1. An eight-lncRNA (SLC25A30-AS1, HPN-AS1, LINC00607, USP2-AS1, HCG20, LINC00638, MKLN1-AS and LINC00652) prognostic score model was constructed for HCC, which was highly associated with TMB and immune infiltration. Next, we constructed a ceRNA network, LINC00638/miR-4732-3p/ULBP1, that may be responsible for NK cell infiltration in HCC with high TMB. However, patients with high ULBP1 possessed a poorer prognosis. Using multiplex immunofluorescence, we found a significant correlation between ULBP1 and PD-L1 in HCC, and patients with high ULBP1 and PD-L1 had the worst prognosis. In brief, the eight-lncRNA model is a reliable tool to predict the prognosis of HCC patients. The LINC00638/miR-4732-3p/ULBP1 axis may regulate immune escape *via* PD-L1 in HCC with high TMB.

## Introduction

Immunotherapy, especially immune checkpoint therapy (ICT), has brought the cancer treatment breakthrough into a brand-new era, and hepatocellular carcinoma (HCC) therapy is the major constituent of the cancer burden globally (1–3). Unfortunately, the response rates of patients to ICT are of great tumor type or individual heterogeneity, and programmed cell death-ligand 1 (PD-L1) expression greatly contributes to patients’ high response to ICT (4). However, responses in patients with HCC to ICT were observed regardless of PD-L1 expression, although the response rate was raised in patients with at least 1% of tumor cells expressing PD-L1 (2). A detailed understanding of the mechanism underlying immune infiltration may favor precision immunotherapy in HCC. Tumor mutation burden (TMB) refers to the total number of mutations in the coding region of the evaluated gene in the tumor cell (TC) genome, which is calculated by the total number of mutations per megabase in the genome (5–7). Previous reports suggested the close association of TMB with immune infiltration, while the molecular mechanism remains inconclusive (5–7).

The past few decades have witnessed the rapid development of long noncoding RNA (lncRNA), a nucleotide sequence longer than 200 nt, in a wide range of fields, including cancers (8). These sequences lack protein-coding potential and can bind to microRNAs (miRNAs) *via* response elements, which affects the expression of miRNA-targeted genes (e.g., a sponge interaction). This phenomenon is also known as the competitive endogenous RNA (ceRNA) hypothesis (9). This mode of action and others, such as sustaining proliferation, cell immortality, angiogenesis, immune evasion and deregulating metabolism, confer lncRNAs a significant role in cancer pathophysiology (8, 10, 11). As a result, a dysregulated lncRNA-related ceRNA network also greatly contributes to the formation and development of HCC (12). For instance, lnc-APUE binds to miR-20b and thereby abolishes its inhibition of E2F1 expression and in turn boots cell cycle progression and cell growth in HCC (13). MCM3AP-AS1 plays an oncogenic role by suppressing miR-194-5p and subsequently upregulates FOXA1 expression in HCC (14). The ceRNA network of HOXD-AS1/miR-130a-3p/SOX4 as well as CASC2/miR-367/FBXW7 may play a prometastatic role in HCC (15, 16). Moreover, several ceRNA networks have been screened as prognostic signatures for HCC, but further experiments to validate these networks are needed in these studies (17, 18). Most importantly, few studies have reported the relationship among the ceRNA network, TMB and immune infiltration in HCC.

In the present study, HCC-related gene expression data were obtained from The Cancer Genome Atlas (TCGA) database and ArrayExpress database (19). First, the differentially expressed lncRNAs (DElncRNAs) in HCC were screened by applying bioinformatic methods. Second, we identified an eight-lncRNA prognostic score for HCC and assessed its correlation with prognosis, TMB and immune infiltration. Furthermore, the ceRNA network LINC00638/miR-4732-3p/ULBP1 (human UL16-binding protein 1) was established in HCC. Finally, we discussed the mechanism of ULBP1-regulated immune escape.

## Methods

### Data Source and Differentially Expressed RNAs

HCC RNA sequencing (RNA-seq) data (50 normal and 373 tumor) were downloaded from TCGA database (https://gdc-portal.nci.nih.gov/), among which 8 tumor RNA-seq data were excluded for no related clinical information. Another dataset, E-TABM-36 (41 tumors), with clinical parameters was downloaded from the ArrayExpress database (https://www.ebi.ac.uk/arrayexpress/) (20). Data from TCGA and ArrayExpress database were annotated by using Transcript ID from Illumina HiSeq 2000 RNA Sequencing and Affymetrix GeneChip Human Genome HG-U133A from Ensembl genome browser 96 database (http://asia.ensembl.org/index.html) (21), respectively. The “limma” package (Version 3.34.7) of R (version 3.6.1) was employed for screening DElncRNAs, and the thresholds were set as |log2-fold change (FC)| > 1.0 and false discovery rate (FDR) < 0.05 (22).

### Identification of lncRNAs With Prognostic Signature

To mine survival-related lncRNAs, univariate Cox analysis achieved by the “survival” package (Version 2.41-1) of R was utilized to investigate the correlation between DElncRNA expression and patient overall survival (OS), with p < 0.05 as a cutoff value (23). After filtration, the least absolute shrinkage and selection operator (LASSO) and support vector machine-recursive feature elimination (SVM-RFE) algorithm were applied to determine the candidate lncRNAs with prognostic signatures (24, 25). The overlapping items shared by the two algorithms were considered to be candidate DElncRNAs for HCC. Subsequently, multivariate Cox analysis was used for final identification of the hub DElncRNAs with prognostic signatures. LncRNAs with p < 0.05 were viewed as the final prognostic indicators in this multivariate analysis.

### Construction of Prognostic Model and Nomogram

According to the multivariate Cox analysis, a prognostic model was constructed as follows: prognostic score (PS) = ∑Coef_lncRNAs_ × Exp_lncRNAs_. Setting the median PS as the cutoff value, patients were divided into low- or high-score groups. After that, this risk score was evaluated by using Kaplan-Meier (K-M) curves and receiver operator characteristic (ROC) analysis in both the TCGA training cohort and E-TABM-36 validation cohort. In addition, univariate and multivariate Cox analyses proceeded to analyze the clinical features and PS, with p < 0.05 as the threshold. To determine the relationship between independent prognostic features and PS, TCGA data were stratified by these clinical features, in which the efficiency of PS was tested. Finally, the nomogram was constructed by variables with significant differences in the above multivariate analysis using the “rms” package (Version 5.1-2) of R, which included lncRNA-based PS and pathologic stage (26).

### TMB Analysis

The “maftools” package (Version 2.6.05) of R was employed to calculate the TMB of HCC (27). The median value of TMB was defined as the cutoff value, and therefore patients were separated into high- or low-TMB groups. The “survival” package was used to mine the correlation between TMB and patient OS. Furthermore, we calculated the TMB value in the abovementioned two risk groups and deduced the correlation between PS and TMB.

### Immune Infiltration Analysis in Prognostic Score Groups

The CIBERSORT algorithm was used to quantify the proportions of immune cells in low- and high-risk groups (28). The “estimate” package of R was applied to infer the fraction of stromal and immune cells in tumor samples and thereby determined the ESTIMATE score, immune score and stromal score in the two groups (29).

### Establishment of ceRNA Network

To construct the lncRNA-miRNA-mRNA network, miRNA and mRNA expression data were downloaded from the TCGA database. The differentially expressed miRNAs (DEmiRNAs) and mRNAs (DEmRNAs) between the low- and high-score groups were explored by the “limma” package of R (22). Afterwards, the DElncRNA-DEmiRNA interaction was obtained from the DIANA-LncBasev2 database (30), and the DEmiRNA-DEmRNA interaction was obtained from the starBase (Version 2.0) database (31). The ceRNA network was constructed and subsequently visualized by Cytoscape (Version 3.6.1) software (32). Finally, Kyoto Encyclopedia of Genes and Genomes (KEGG) analysis of the related mRNAs was realized by using the DAVID 6.8 online tool (33, 34).

### Cell Lines and Culture

The human HCC cell lines PLC/PRF/5 and MHCC97H were maintained at the Liver Cancer Institute of Zhongshan Hospital, Fudan University (Shanghai, China). Both cell lines were maintained in Dulbecco’s modified Eagle medium (DMEM) (Invitrogen) with 10% fetal bovine serum (FBS) (Gibco) and 1% penicillin-streptomycin (Gibco) at 37°C in a humidified incubator supplemented with 5% CO_2_.

### Fluorescence *In Situ* Hybridization (FISH)

PLC/PRF/5 and MHCC97H cells were cultured in glass chamber slides, fixed with 4% paraformaldehyde, and permeated with 0.1% Triton X-100. After washing and treating with pre-hybridization buffer, hybridization was carried out by using a FISH detection kit (Ribo Biotechnology Co., Ltd.) including probes for LINC00638 (GCTCCGTAGCCTATTCACCCCCACCAGACCCTT) and miR-4732-3p (CAGAACAGGACAGGTCAGGGC). The nuclei were stained with 4′,6-diamidino-2-phenylindole and dihydrochloride (DAPI).

### Immunofluorescence

PLC/PRF/5 and MHCC97H cells were fixed with 4% paraformaldehyde, permeabilized with 0.1% Triton X-100, and blocked with 5% bovine serum albumin for 30 min. Then, the cells were incubated with anti-ULBP1 antibody (sc-53131; Santa Cruz Biotechnology, Inc., CA, USA) or anti-PD-L1 antibody (#13684; Cell Signaling Technology, Inc., Beverly, MA, USA) overnight at 4°C and subsequently with the related secondary antibody at room temperature for 50 min. The cells were observed under a fluorescence microscope (Olympus, Tokyo, Japan).

### Luciferase Reporter Assay

The potential binding site between miR-4732-3p and LINC00638 or ULBP1 was predicted, and a related mutant was designed. LINC00638-WT, LINC00638-MUT, ULBP1-WT and ULBP1-MUT were cloned into the luciferase plasmids separately. Then, these plasmids and miR-4732-3p were cotransfected into HEK293 cells. After incubation for 48 h, the luciferase activity was detected by the Dual-Luciferase^®^ Reporter Assay System (Promega, Madison, WI, USA).

### Co-Immunoprecipitation (Co-IP)

The interaction between ULBP1 and PD-L1 was estimated by using Co-IP. Briefly, the protein was exacted from PLC/PRF/5 and MHCC97H cells, and then incubated with antibodies for ULBP1, PD-L1 or IgG overnight. Protein A/G-agarose beads were followed added to the complex and incubated for 4 h. Western blot assay was employed to detect the level of ULBP1 and PD-L1.

### Patients and Tissue Microarrays

Tumor specimens and matched nontumor liver tissues (adjacent to the tumor within a distance of 10 mm) were collected from 336 HCC patients who underwent hepatectomy at Zhongshan Hospital of Fudan University from 2005 to 2012. The tumor tissue microarrays (TMAs; n = 316) were constructed based on two cores taken from representative tissue areas, which was described previously (35). The related clinical features of all patients were recorded simultaneously. Informed consent was also acquired from the involved patients, and the study was approved by the Zhongshan Hospital Ethics Committee.

### Multiplex Immunofluorescence

Multiplex immunofluorescence (mIF) was employed to detect the expression of ULBP1, PD-L1, CD56 and cytokeratin 18 (CK18) in HCC. First, the slides were deparaffined and rehydrated, and antigen retrieval was performed in Tris-EDTA buffer (pH 9.0) at the boiling point. Second, endogenous peroxidase activity and nonspecific antigens were blocked by 3% hydrogen peroxide and goat serum solution, respectively. Then, the slides were incubated with primary antibody overnight at 4°C, followed by horseradish peroxidase-conjugated secondary antibody at room temperature for 30 min. Next, the slides were treated with Opal tyramide signal amplification Fuorochromes according to the instructions of Opal 7-Color Manual IHC Kit (NEL811001KT; Perkin Elmer, Germany). Between each staining cycle, slides were microwaved to strip the Ab-TSA complex and blocked with goat serum solution. Finally, DAPI was used to stain nuclei, and the nuclei were mounted with glycerine. Anti-PD-L1 antibody (#13684) and anti-CK18 antibody (#4548) were purchased from Cell Signaling Technology, Inc. (Beverly, MA, USA), and anti-CD56 antibody (ab75813) was purchased from Abcam (CA, USA).

### Imaging and Colocalization Analysis of mIF

After staining, the slides were scanned by using Pannoramic DESK/MIDI/250/1000 (3DHISTECH; Hungary). The counts of marker-positive cells, colocalized cells and total cells were quantified by using the Indica Labs-Highplex FL (v3.1.0) module of Halo software (v3.0.311.314) in tumor or peritumor tissues. CK18 and CD56 were employed to identify the populations of TC (CK18^+^CD56^−^) and NK cells (NKc; CD56^−^CK18^+^). Tissues with PD-L1-positive cells of ≥ 5% were considered the high-PD-L1 group (36). X-tile software was applied to calculate the optimal cutoff value of ULBP1-positive cells in HCC (Yale University, New Haven, CT, USA) (37). The cutoff value was 30% in tumor tissues and 10% in peritumor tissues.

### Immunofluorescence Intensity Analysis of mIF

The Indica Labs-Area Quantification FL (v2.1.2) module of Halo software was used to calculate the immunofluorescence intensity of the slides. The optimal cutoff value of immunofluorescence intensity was determined by using X-tile software. For ULBP1, the cutoff value was 38.0 in tumor tissues and 26.9 in peritumor tissues. For PD-L1, the cutoff value was 10.3 in tumor tissues and 15.5 in peritumor tissues.

### Statistics

The t-test was used for comparison, and continuous variables are shown as the mean ± standard deviation (SD). The PS model was constructed using Cox analysis, where the hazard ratio (HR) was the 95% confidence interval (CI). OS analyzed by the log-rank test indicated the time from the date of diagnosis to the date of last follow-up or death. The predictive performance of the PS model was assessed by using nomograms and ROC curves. All statistical analyses were performed using SPSS version 22.0 (IBM SPSS Statistics, Chicago, IL, US) and R software (version 3.5.2). The packages of R were as follows: “limma”, “survival”, “lars”, “caret”, “rms”, “estimate”, and “maftools”. If not specified above, *p* < 0.05 was considered statistically significant.

## Results

### Exploration of Hub lncRNAs With Prognostic Signatures for HCC

**Figure 1:**
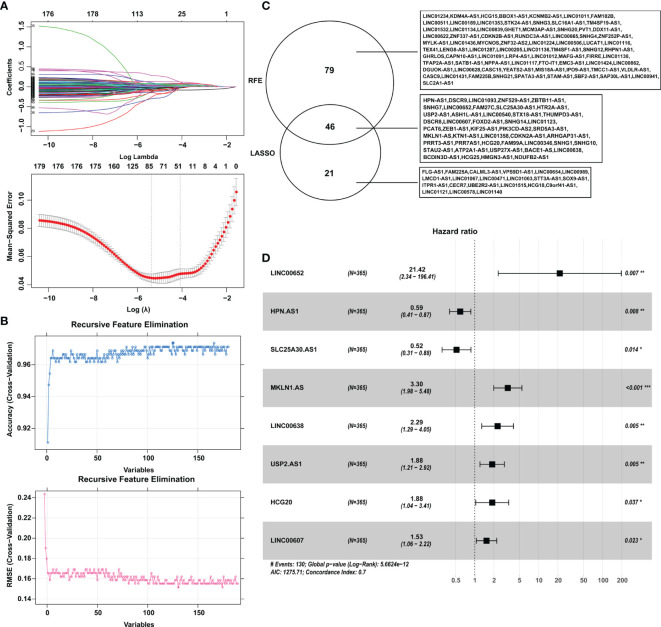
Identification of the hub DElncRNAs with prognostic signatures. **(A)** Parameter diagram of LASSO algorithm. **(B)** Parameter diagram of SVM-RFE algorithm. **(C)** The overlapping DElncRNAs between the LASSO and SVM-RFE algorithms. **(D)** Forrest plot demonstrating the multivariate Cox analysis results of the hub lncRNAs. LASSO, least absolute shrinkage and selection operator; SVM-RFE, support vector machine-recursive feature elimination. *p < 0.05, **p < 0.01, ***p < 0.001.

A total of 615 DElncRNAs were mined between 365 tumor samples and 50 nontumor samples in the training cohort from TCGA according to the cutoff criteria of |log_2_FC| > 1 and FDR < 0.05. Combined with patient survival, 180 DElncRNAs with a significant prognostic signature were selected by using univariate Cox analysis with p < 0.05. Subsequently, LASSO and SVM-RFE algorithms were employed to shrink the number of lncRNAs, and 67 and 125 DElncRNAs were screened, respectively (**Figures 1A–C**). The shared objects of the two algorithms consisted of 46 DElncRNAs (**Figure 1C**). Ultimately, 8 lncRNAs (SLC25A30-AS1, HPN-AS1, LINC00607, USP2-AS1, HCG20, LINC00638, MKLN1-AS and LINC00652) were confirmed as hub DElncRNAs in HCC with prognostic signatures by multivariate Cox analysis (**Figure 1D** and **Table 1**). The K-M curves also validated the significant correlation between these hub lncRNAs and patient OS in the training cohort (**Figure S1**).

**Table 1:** Independent prognostic characteristic of DElncRNAs.

LncRNA	Coefficient	HR	95% CI	SE	Z score	P-value
SLC25A30-AS1	-0.6536	0.5201	0.3087-0.8764	0.2662	-2.455	0.014
HPN-AS1	-0.5201	0.5945	0.4052-0.8722	0.1956	-2.659	0.008
LINC00607	0.4276	1.5336	1.0595-2.2200	0.1887	2.266	0.023
USP2-AS1	0.6304	1.8783	1.2100-2.9158	0.2244	2.81	0.005
HCG20	0.6317	1.8808	1.0385-3.4063	0.303	2.085	0.037
LINC00638	0.8265	2.2852	1.2898-4.0488	0.2918	2.832	0.005
MKLN1-AS	1.1926	3.2958	1.9834-5.4766	0.2591	4.603	<0.001
LINC00652	3.0642	21.4182	2.3356-196.4128	1.1306	2.71	0.007

CI, confidence interval; HR, hazard ratio; SE, standard error.

### Construction of an Eight-lncRNA Prognostic Signature for HCC

**Figure 2:**
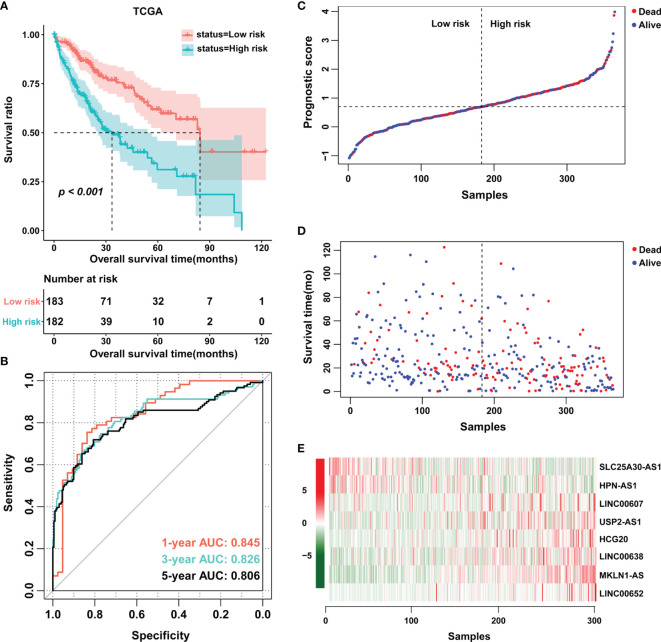
Establishment of an eight-lncRNA prognostic score. **(A)** K-M curves were plotted to evaluate the OS probabilities in the training cohort. **(B)** ROC curves were plotted for 1-, 3- and 5-year OS in the training cohort. **(C**, **D)** Risk survival status plot. **(E)** Heatmap of the expression profiles of members of the eight-lncRNA prognostic score. TCGA, The Cancer Genome Atlas.

Based on the multivariate Cox analysis, a PS formula was established using the following equation: PS = (-0.6536 × Exp SLC25A30-AS1) + (-0.5201 × Exp HPN-AS1) + (0.4276 × Exp LINC00607) + (0.6304 × Exp USP2-AS1) + (0.6317 × Exp HCG20) + (0.8265 × Exp LINC00638) + (1.1926 × Exp MKLN1-AS) + (3.0642 × Exp LINC00652). Then, the PS values of each patient were calculated, whereby the patients were divided into low- or high-score groups according to the median cutoff point. The patients in the high-score group had a shorter OS than those in the low-score group, and the area under the curve (AUC) values of the ROC curve for PS were 0.845, 0.826 and 0.806 for 1-year survival, 3-year survival and 5-year survival, respectively (**Figures 2A, B**). Of note, an increase in cancer-related death and a decrease in patient survival were observed with increasing PS values, and the expression level of each related lncRNA is shown in the heatmap (**Figures 2C–E**). In addition, the prognostic model was also estimated in the validation cohort E-TABM-36, in which similar encouraging results were observed (**Figure S2**). These data indicated the potential predictive effect of PS in patients with HCC.

### The Significant Correlation Between PS and OS in Stratified Analysis

**Figure 3:**
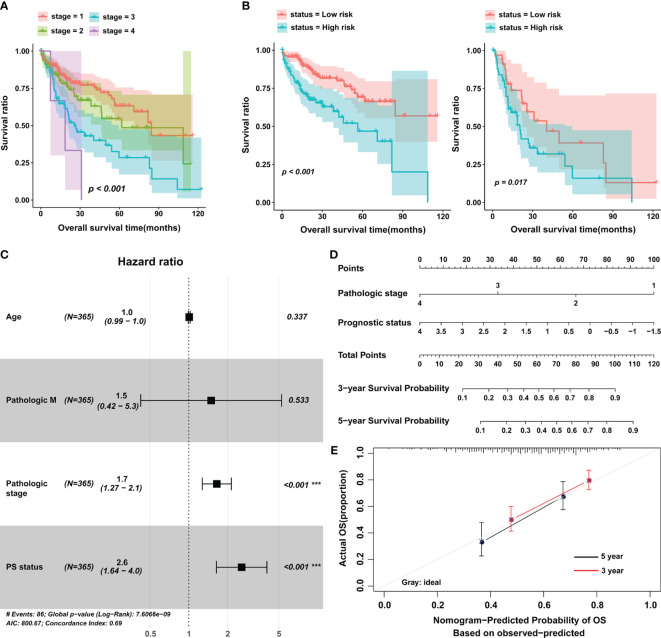
The prognostic value of the eight-lncRNA signature in stratified analysis and construction of nomogram. **(A)** K-M curves were plotted to evaluate the OS probabilities for different pathological stages. **(B)** Stage I/II group (left) and stage III/IV group (right). **(C)** Multivariate Cox analysis in the training cohort. **(D)** Nomogram to predict the 3- and 5-year OS in patients with HCC. **(E)** Calibration curve for the nomogram model in the training cohort. The gray line represents the ideal nomogram, and the red line and black line represent the 3-year and 5-year observed nomograms. The horizontal axis represents the nomogram-predicted 5-year survival rate, and the vertical axis represents the actual 5-year survival rate. PS, prognostic score; OS, overall survival. ***p < 0.001.

Next, we investigated the influence of clinical features on the predictive value of PS. Data from univariate and multivariate analyses suggested that PS and pathologic stage were independent prognostic factors for HCC (**Figure 3A** and **Table 2**). Setting pathologic stage as a stratification factor, PS remained a significant prognostic signature for patients with HCC (**Figure 3B**). The results of multivariate analysis were also shown by a forest plot (**Figure 3C**). To improve the clinical application of PS, a nomogram was constructed based on it as well as pathologic stage (**Figure 3D**). Data from the calibration plots suggested that the 3-year and 5-year OS rates in patients with HCC were predicted well when compared with an ideal model in the training cohort (**Figure 3E**).

**Table 2:** Univariate and multivariate analyses of the prognostic model.

Clinical characteristics	Uni-variable cox		Multi-variable cox	
	HR (95% CI)	P value	HR (95% CI)	P value
Age (years, mean ± SD)	1.012 (0.998-1.026)	**0.008**	1.008 (0.992-1.025)	0.337
Gender (Male/Female)	0.817 (0.573-1.164)	0.262	–	–
Pathologic M (M0/M1/-)	4.032 (1.267-12.83)	**0.011**	1.493 (0.424-5.256)	0.533
Pathologic N (N0/N1/-)	2.004 (0.491-8.181)	0.333	–	–
Pathologic T (T1/T2/T3/T4/-)	1.675 (1.397-2.007)	0.102	–	–
Pathologic stage (I/II/III/IV/-)	1.661 (1.355-2.037)	**<0.001**	1.651 (1.274-2.141)	**<0.001**
Histologic grade (G1/G2/G3/G4)	1.121 (0.887-1.416)	0.339	–	–
Vascular invasion (Yes/No/-)	1.351 (0.892-2.047)	0.154	–	–
Recurrence (Yes/No/-)	1.375 (0.914-2.068)	0.125	–	–
PS status (High/Low)	2.593 (1.806-3.722)	**<0.001**	2.576 (1.641-4.043)	**<0.001**

CI, confidence interval; HR, hazard ratio; SD, standard deviation; PS, prognostic score.

The bold type represents statistical differences.

### The PS Was Associated With TMB

**Figure 4:**
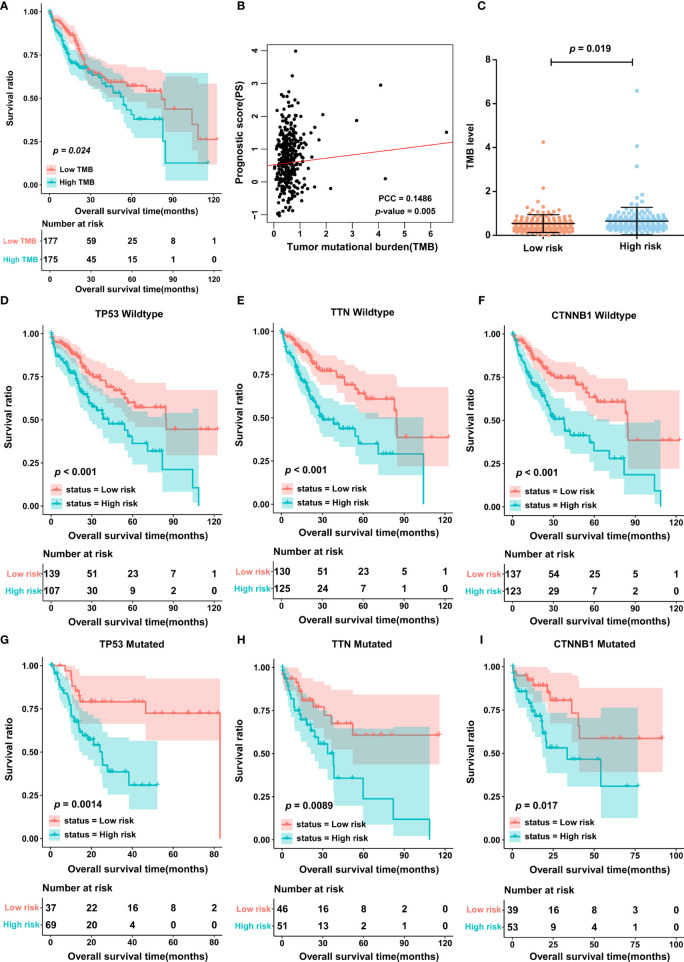
The TMB between low- and high-score group. **(A)** K-M curves displaying the association of TMB and OS in patients with HCC. **(B)** The correlation between PS and TMB was analyzed by using Pearson correlation analysis. PCC, Pearson correlation coefficient. **(C)** TMB level in low- and high-score group. **(D–I)** The correlation of PS and OS in different stratified groups, including the *TP53* wild-type group **(D)**, *TTN* wild-type group **(E)**, *CTNNB1* wild-type group **(F)**, *TP53* mutation group **(G)**, *TTN* mutation group **(H)**, and *CTNNB1* mutation group **(I)**. TMB, tumor mutation burden.

In light of the importance of TMB in cancer, we also estimated the correlation between PS and TMB in the TCGA cohort. First, patients were divided into two groups, the high- and low-TMB groups, based on the median cutoff point of the TMB value. The results of K-M curves indicated that patients in the high-TMB group possessed a significantly lower OS rate than those in the low-TMB group (**Figure 4A**). Most importantly, TMB status was significantly associated with PS, and in other words, patients in the high-score group may have also had a high level of TMB (**Figures 4B, C**). In addition, the 3 most frequently mutated genes in HCC were employed as stratification factors, and therefore, the correlation between PS and OS was further investigated in the TCGA cohort^6^. The data showed that in the cohort of patients with wild-type or mutant *TP53*, wild-type or mutant *TTN*, and wild-type or mutant *CTNNB1*, PS still possessed clinically and statistically significant value for predicting patient prognosis (**Figures 4D–I**).

### Entanglement Among the PS, TMB and Immune Infiltration

**Figure 5:**
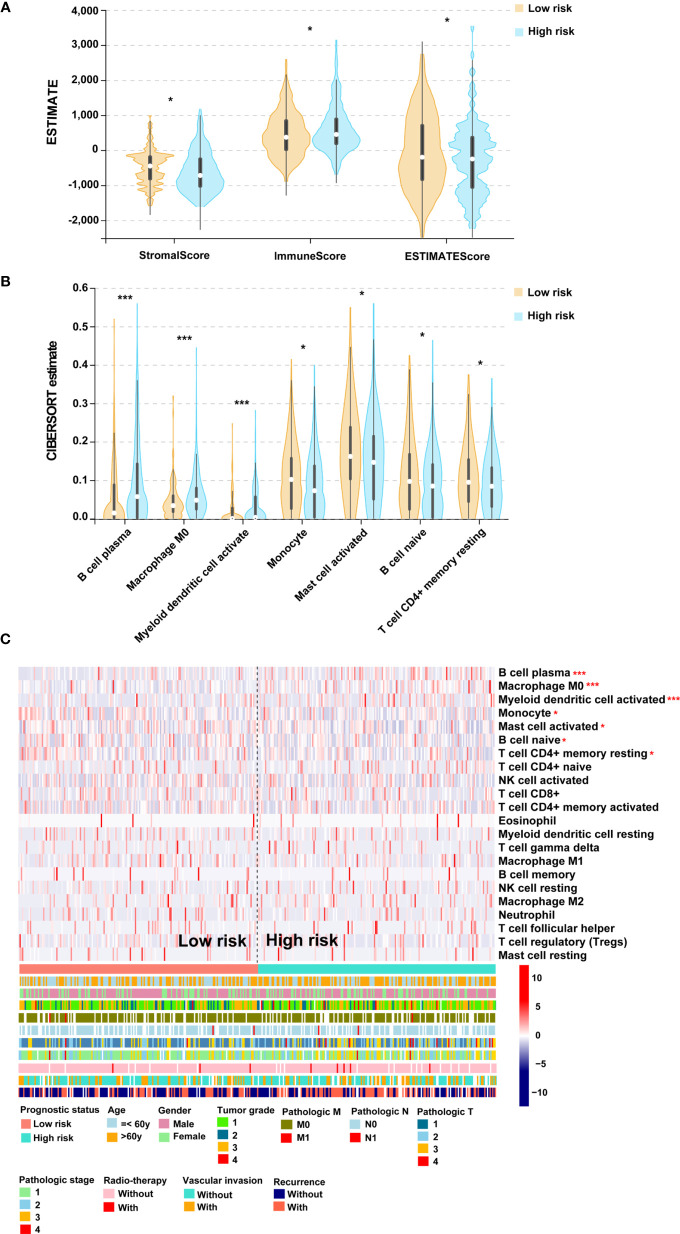
The association of PS and immune infiltration. **(A)** Violin plot for the results of ESTIMATE algorithm. **(B)** Violin plot for the results of CIBERSORT algorithm. **(C)** Heatmap for immune responses based on the CIBERSORT algorithm among the low- and high-score groups. PS, prognostic score. *p < 0.05, ***p < 0.001.

To determine the association of PS with immune infiltration, ESTIMATE and CIBERSORT algorithms were processed to estimate the association in the TCGA cohort. After estimation by the ESTIMATE algorithm, a higher ESTIMATE score was observed in the low-score cohort (**Figure 5A**). A fraction of stromal cells was associated with the low-score cohort, while a fraction of immune cells was associated with the high-score cohort (**Figure 5A**). The results of the CIBERSORT algorithm indicated that B cell plasma, M0 microphages and myeloid dendritic cell activation were positively correlated with PS, but monocytes, activated mast cells, naïve B cells and resting CD4+ memory T cells were negatively correlated with PS (**Figures 5B, C**). Taken together, these results suggested a high level of immune infiltration in patients in the high-score group. We then analyzed the second-generation sequencing (NGS) data of 20 patients from a real-world study. According to the TMB value, the top 10 patients were classified into the high-TMB group. The data showed that 1 case with high expression of PD-L1, 1 case with microsatellite instability-high (MSI-H), and 1 case with both high expression of PD-L1 and MSI-H were observed in the high-TMB group (**Figure S3**). From the above, patients in the high-score group may possess high immune infiltration and high TMB.

### Establishment of a ceRNA Network

**Figure 6:**
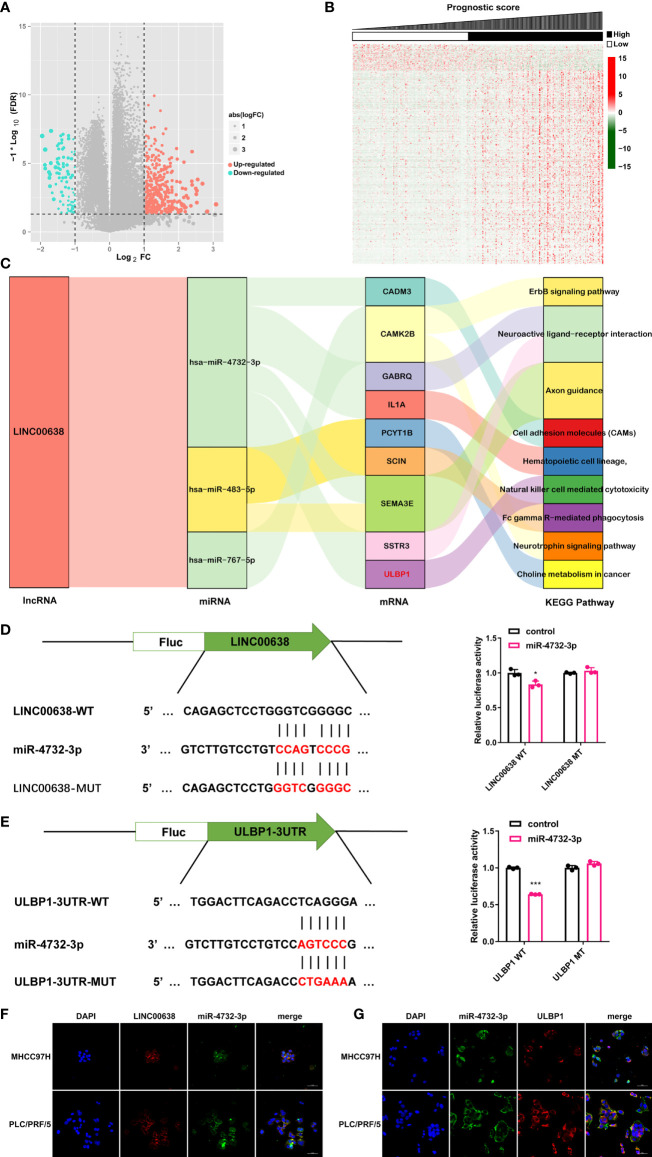
ceRNA network. **(A, B)** Volcano plot **(A)** and heatmap **(B)** of the DEmiRNAs and DEmRNAs. **(C)** Sankey plot demonstrating the ceRNA network of LINC000638 and KEGG results of related mRNAs. **(D)** Schematic illustration of the binding site of LINC00638 with miR-4732-3p (left). The luciferase activity of LINC00638-WT/LINC00638-MUT in HEK293 cells cotransfected with miR-4732-3p mimics was detected by using a luciferase reporter assay (right). **(E)** Schematic illustration of the binding site of ULBP1 with miR-4732-3p (left). The luciferase activity of ULBP1-WT/ULBP1-MUT in HEK293 cells cotransfected with miR-4732-3p mimics was measured by using a luciferase reporter assay (right). **(F, G)**. The colocalization of miR-4732-3p and LINC000638 **(F)** or ULBP1 **(G)** was observed in HCC cell lines. FC, fold change. *p < 0.05, ***p < 0.001.

The ceRNA network hypothesis is primarily responsible for the function of lncRNAs (8). To establish a ceRNA network, 7 DEmiRNAs and 469 DEmRNAs were identified between the low- and high-score groups based on PS (**Figures 6A, B**). Considering the negative correlation between miRNA and mRNA expression, 5 lncRNA-miRNA interactions and 135 miRNA-mRNA interactions were obtained by using DIANA-LncBasev2 and the starBase database and visualized by using Cytoscape software (**Figure S4**). Of note, LINC00638 was involved in the majority of miRNAs as well as mRNAs and was therefore considered the key lncRNA (**Figure S4**). In addition, these lncRNA signatures were tightly connected with immune infiltration and TMB. We then analyzed the function of the mRNAs regulated by these lncRNAs and found that they were mainly enriched in 8 signaling pathways. Among these pathways, the natural killer cell-mediated cytotoxicity pathway had a high correlation with immune infiltration (**Figure 6C**). Taken together, only the ceRNA network, LINC00638/miR-4732-3p/ULBP1, met our requirement and became the next study focus (**Figure 6C**). After that, the binding position between miR-4732-3p and LINC00638 or ULBP1 was displayed (**Figures 6D, E**). The luciferase reporter assay indicated that miR-4732-3p significantly decreased the luciferase activity of LINC00638-WT and ULBP1-WT but not LINC00638-MUT or ULBP1-MUT (**Figures 6D, E**). In addition, colocalization analysis showed that LINC00638 and miR-4732-3p colocalized in the HCC cell lines MHCC97H and PLC/PRF/5, as did miR-4732-3p and ULBP1 (**Figures 6F, G**). These data suggested that LINC00638 may act as a sponge for miR-4732-3p and remove its inhibition of ULBP1 expression.

### PD-L1 Contributed to Immune Escape in HCC With High ULBP1

**Figure 7:**
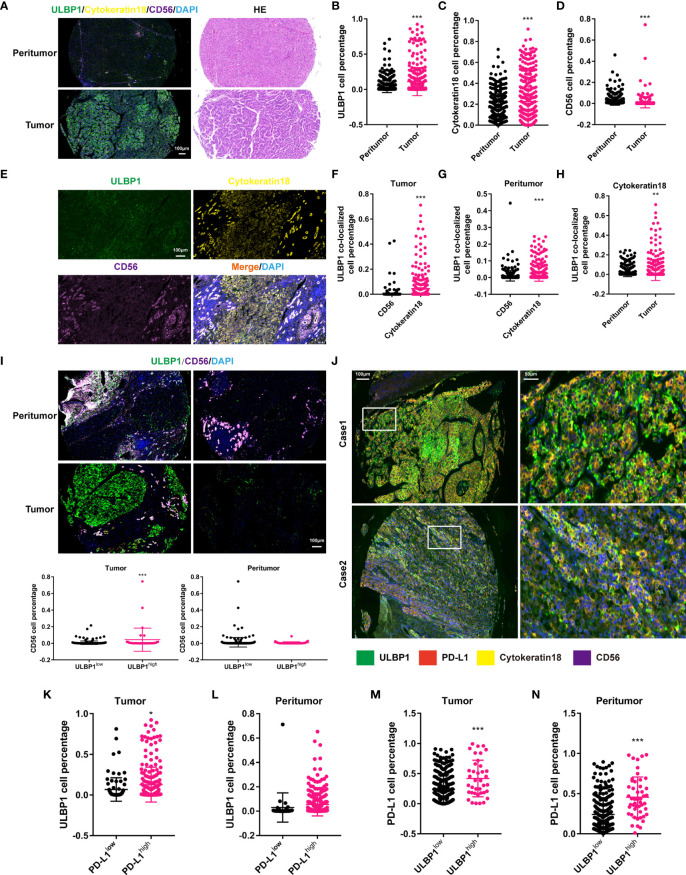
Correlation between ULBP1^+^ cells and PD-L1^+^ cells in HCC. **(A)** The expression of ULBP1 in HCC (left). HE staining of tumor and peritumor tissues in HCC (right). **(B–D)** The proportion of ULBP1^+^ **(B)**, CK18^+^ **(C)** and CD56^+^ **(D)** cells in HCC. ULBP1^+^ and CK18^+^ cells were more distributed in tumor tissues, while CD56^+^ cells were more distributed in peritumor tissues. **(E)** Colocalization ULBP1, CK18 and CD56 in HCC. **(F, G)** The proportion of ULBP1^+^CD56^+^ cells was much less than that of ULBP1^+^CK18^+^ cells in tumor tissues **(F)** or peritumor tissues **(G)**. **(H)** The proportion of ULBP1^+^CK18^+^ cells was significantly higher in tumor tissues than in peritumor tissues. **(I)** The proportion of CD56^+^ cells in the low ULBP1 group was lower than that in the high ULBP1 group in tumor tissues, but no significant difference was observed in peritumor tissues. **(J)** Colocalization of ULBP1, PD-L1, CK18 and CD56 in HCC. **(K, L)** The proportion of ULBP1^+^ cells in the low or high PD-L1 group in tumor **(K)** and peritumor tissues **(L)**. **(M, N)** The proportion of PD-L1^+^ cells in the low or high ULBP1 group in tumor **(M)** and peritumor tissues **(N)**. ^*^ *p* < 0.05, ^**^ *p* < 0.01 and ^***^ *p* < 0.001. HE, hematoxylin and eosin.

**Figure 8:**
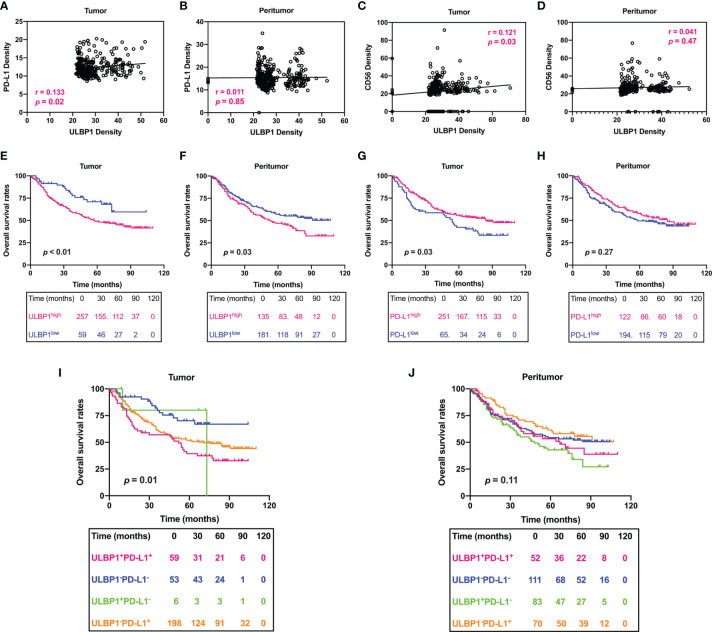
Prognostic signature of the density of ULBP1 and PD-L1 in HCC. **(A–D)** The association between the densities of PD-L1, CD56 and ULBP1 was analyzed by Pearson correlation analysis. **(E–H)** Survival analysis of ULBP1 or PD-L1 in HCC. **(I, J)** Combined survival analysis of ULBP1 and PD-L1 in HCC.

In view of the association between ULBP1 and NKc (38), we investigated this association in HCC by using mIF staining. The proportion of ULBP1^+^ cells, TC or NKc had a significant increase in tumor tissues compared with peritumor tissues (**Figures 7A–D**). Colocalized analysis showed that the number of ULBP1^+^CK18^+^ cells was far greater than that of ULBP1^+^CD56^+^ cells in tumor or peritumor tissues, and ULBP1^+^CK18^+^ cells had a remarkable enrichment in tumor tissues (**Figures 7E–H**), indicating that ULBP1 was mainly located in TC. Of note, NKc had a significant reduction in the low ULBP1 group compared to the high ULBP1 group in tumor tissues, while no significant difference was observed in peritumor tissues (**Figure 7I**). This result indicated that ULBP1 could promote the infiltration of NKc into tumors, meaning that HCC with high ULBP1 may possess favorable outcomes. However, a previous study showed that ULBP1 was associated with a poor prognosis in HCC (39), inconsistent with the above statement. To address this contradiction, we hypothesized that some immune inhibitory factors interact with ULBP1 in HCC. Our data found that PD-L1, a well-known inhibitory factor of NKc (40), was expressed in the majority of peritumor tissues (88.92%, 281/316) or tumor tissues (72.15%, 228/316) (**Figure S5**). The colocalization of ULBP1 and PD-L1 was also observed in the TC of tumor tissues (**Figure 7J**), which was also confirmed in the HCC cell lines MHCC97H and PLC/PRF/5 (**Figure S6**). In addition, we validated the relationship between ULBP1 and PD-L1 by co-immunoprecipitation (Co-IP). Endogenous ULBP1 was co-immunoprecipitated by PD-L1 antibodies whereas PD-L1 were reciprocally co-immunoprecipitated by the ULBP1 antibody in MHCC97H and PLC/PRF/5 cells (**Figure S7**). Further analysis revealed that the high PD-L1 group in tumor tissues had more ULBP1^+^ cells than the low PD-L1 group (*p* < 0.05), while no significant change was observed in peritumor tissues (**Figures 7K, L**). In turn, the high ULBP1 group in both tumor tissues (*p* < 0.001) and peritumor tissues (*p* < 0.001) had more PD-L1^+^ cells than the low ULBP1 group (**Figures 7M, N**). To accurately confirm the correlation between ULBP1 and PD-L1 or CD56, their immunofluorescence intensity was analyzed. ULBP1 was positively correlated with PD-L1 and CD56 in tumor tissues but not in peritumor tissues (**Figures 8A–D**). These results indicated that ULBP1 in HCC tumor tissues could recruit NK cells to tumors accompanied by PD-L1 expression. In summary, PD-L1 may account for immune escape in HCC with high ULBP1.

### Combined Survival Analysis of ULBP1 and PD-L1 in HCC

Finally, the correlation between patient OS and ULBP1 or PD-L1 was evaluated by using a survival curve. Patients were divided into ULBP1^high^/ULBP1^low^ groups or PD-L1^high^/PD-L1^low^ groups according to the previously set cutoff value. HCC patients with high ULBP1 in tumor tissues had a significantly shorter OS than those with low ULBP1 (*p* < 0.01), and ULBP1 in peritumor tissues was also significantly related to the OS of patients (*p* = 0.03) (**Figures 8E, F**). In addition, patients with high PD-L1 in tumor tissues had a significantly longer OS than those with low PD-L1 (*p* = 0.03), but PD-L1 in peritumor tissues also had no prognostic significance (*p* = 0.27) (**Figures 8G, H**). Combined survival analysis indicated that patients with high ULBP1 and high PD-L1 in tumor tissues had the worst prognosis, while patients with low ULBP1 and low PD-L1 had the best prognosis (**Figure 8I**). Of note, ULBP1 and PD-L1 in the peritumor tissues had no prognostic significance in the combined analysis (**Figure 8J**).

## Discussion

In this paper, we identified a set of lncRNAs with independent prognostic signatures and constructed an eight-lncRNA PS model for HCC according to combined database analysis. We found that this PS model had a high correlation with immune infiltration and TMB. In addition, we established an immune-related ceRNA network, LINC00638/miR-4732-3p/ULBP1, in HCC. This ceRNA network may be a mechanism for immune escape in HCC with high TMB.

Recent studies set up several prognostic risk models with lncRNAs aimed at predicting the prognosis of patients with HCC *via* bioinformatics analysis (17, 18, 41). These papers demonstrated the potential clinical value of lncRNAs for predicting HCC patients’ OS. Therefore, we established an eight-lncRNA prognostic risk model *via* diversified algorithms and regression analysis, which was also significantly related to several stratification groups in HCC. K-M curves showed that all 8 lncRNAs could serve as independent prognostic factors for HCC. Among these biomarkers, only MKLN1-AS has been reported in HCC and is considered a diagnostic and prognostic biomarker (42). HPN-AS1 could be utilized in the diagnosis of prostate cancer (43); LINC00607 facilitates the proliferation and invasion of osteosarcoma (44); USP2-AS1 promotes the progression of colon cancer and ovarian cancer (45, 46); and LINC00638 and LINC00652 have been reported in nontumor diseases (47–50), while no study has revealed the function of the remaining lncRNAs. As a result, further exploration of these lncRNAs may improve insight into the molecules underlying HCC.

The relationship between TMB and response to ICT is an effective supplement for precision immunotherapy in cancers (51). A significant correlation was observed in various cancer types, including nonsmall cell lung cancer (NSCLC), small cell lung cancer, melanoma, colorectal cancer, renal cell cancer, and urothelial carcinoma; among these cancer types, the study on NSCLC is at the leading edge (52). The higher the level of TMB a tumor has, the more neoantigens it is also likely to yield, accounting for its influence on the tumor’s response to ICT (51). Unfortunately, however, few clinical studies have revealed the role of TMB in HCC. We and others found a significant correlation between TMB and immune infiltration in HCC (5–7). Most importantly, the PS model was significantly associated with TMB and immune infiltration. In the high-score group, high TMB and immune infiltration were also observed, indicating that these hub lncRNAs may establish some connection between TMB and immune infiltration in HCC. Considering that result, focusing on these hub lncRNAs may be conducive to understanding the underlying mechanism of the intricate connection between immune infiltration and TMB, as well as achieving precision immunotherapy.

Compelling evidence suggests that the ceRNA hypothesis is the primary mechanism for assigning lncRNA function (9). Multiple lncRNA-miRNA-mRNA networks have been reported to concern various HCC tumor hallmarks, such as cell proliferation, epithelial-mesenchymal transition, metastasis and chemoresistance (53). Here, we constructed a novel ceRNA network, LINC00638/miR-4732-3p/ULBP1, for HCC. Colocalization analysis and a luciferase assay were employed to verify the ceRNA regulatory network. KEGG analysis indicated the association of the ceRNA network with NKc in HCC, suggesting that it may account for the correlation between TMB and immune infiltration. Previous studies indicated that LINC00638 mainly focused on nontumor diseases, such as rheumatoid arthritis, lumbar disc degeneration and neonatal sepsis (47–49); miR-4732-3p is a grade-related biomarker for prostate cancer (54). No studies have revealed the interaction between LINC00638 and miR-4732-3p in HCC, let alone this established ceRNA. As a result, this ceRNA network was first reported in HCC, and further research is warranted.

The mRNA in the network, ULBP1, belongs to the extended MHC class I-like family and acts as one of the major ligands of activatory receptor natural killer cell group 2, member D (NKG2D), which is constitutively expressed on NKc cells, most NKT cells, some γδ T cells and CD8 T cells (55). We and others found that ULBP1 is upregulated in HCC tissues and associated with poor prognosis (39). Data from mIF showed that high ULBP1 expression brings out high NKc infiltration in HCC. However, it is notable that ULBP1 is an NKc activator and could recruit NKc and T cells to the tumor, which can eliminate tumors directly (38, 56). The function of ULBP1 seems to contradict its prognostic value in HCC. Hence, we inferred that other immune inhibitory factors may participate in HCC with high ULBP1. A previous study was indicative of PD-L1-mediated suppression of tumor-infiltrating NKc (40). Inspired by that study, we determined the expression of PD-L1 in tumor and peritumor tissues and found high PD-L1 expression in the majority of HCC samples. Colocalization analysis revealed that ULBP1 and PD-L1 are coexpressed in HCC, as proven in HCC tissues and cell lines. Pearson correlation analysis suggested that a significant association of ULBP1 with PD-L1 or CD56 was observed in tumor tissues but not in peritumor tissues. These data indicated that ULBP1 in tumor tissues can attract the infiltration of NKc to tumors, while concomitant PD-L1 restricts its activity. Of note, the ULBP1-related ceRNA network is closely related to TMB in HCC, and ULBP1-related PD-L1 expression may also be the mechanism of immune escape in HCC with high TMB.

Combined survival analysis showed that only ULBP1 and PD-L1 in tumor tissues possessed a significant prognostic signature for patients with HCC. Previous data on the prognostic role of PD-L1 in HCC were inconsistent: some studies supported that PD-L1 is correlated with a poor prognosis in HCC (57–59), whereas other studies opposed (60, 61). Here, the patients fell into the PD-L1^high^ and PD-L1^low^ groups according to the cutoff value of immunofluorescence intensity calculated by X-tile software. We found that low PD-L1 in tumor tissues predicted a shorter patient OS than high PD-L1. The varied detection and analysis methods may account for great heterogeneity among the different studies. In addition, combined survival analysis suggested that patients with high ULBP1 and high PD-L1 had a shorter OS than those with high ULBP1 and low PD-L1. Therefore, PD-L1 may be responsible for the poor prognosis of HCC patients with high ULBP1.

In summary, an eight-lncRNA PS model was established, and this PS model not only has a significant correlation with patient OS but also with TMB and immune infiltration. We focused on one lncRNA and thereby constructed an immune-related ceRNA network, LINC00638/miR-4732-3p/ULBP1, which may be a potential mechanism for the association between TMB and immune infiltration. In addition, we revealed that tumors with high ULBP1 may possess high PD-L1 expression. This relationship may explain why patients with high ULBP1 expression have a poor prognosis. Hence, further study on this ceRNA network may contribute to the development of ICT in HCC, and targeting ULBP1 may also provide a potential treatment strategy for HCC.

## Data Availability Statement

The original contributions presented in the study are included in the article/**Supplementary Material**. Further inquiries can be directed to the corresponding authors.

## Ethics Statement

The studies involving human participants were reviewed and approved by the Zhongshan Hospital Ethics Committee. The patients/participants provided their written informed consent to participate in this study.

## Author Contributions

Conceptualization: FQ and JX. Biological experiments and sequencing: FQ, XD, WQ, DZ, MH, and YB. Clinical experimental analyses and statistical evaluations: BY, FQ, and ZZ. Statistical and bioinformatic analyses: FQ, XD, and ZZ. Writing – original draft: FQ and XD. Writing – review and editing: BY, WQ, and JX. All authors contributed to the article and approved the submitted version.

## Funding

This study was supported by the National Natural Science Foundation of China (grant nos. 81772590 and 81572395).

## Conflict of Interest

Author DZ, MH, and YB were employed by the company of 3D Medicines Inc.

The remaining authors declare that the research was conducted in the absence of any commercial or financial relationships that could be construed as a potential conflict of interest.

## Publisher’s Note

All claims expressed in this article are solely those of the authors and do not necessarily represent those of their affiliated organizations, or those of the publisher, the editors and the reviewers. Any product that may be evaluated in this article, or claim that may be made by its manufacturer, is not guaranteed or endorsed by the publisher.
