# Supplementary material for: Tumor Mutation Burden-Associated LINC00638/miR-4732-3p/ULBP1 Axis Promotes Immune Escape via PD-L1 in Hepatocellular Carcinoma
Source: Front Oncol. 2021 Sep 8;11:729340. doi: 10.3389/fonc.2021.729340 (PMC8456090; doi:10.3389/fonc.2021.729340)
Supplement: Supplementary file 1 [file DataSheet_1.docx]

**Supplementary Material**

**Tumor mutation burden associated LINC00638/miR-4732-3p/ULBP1 axis promotes immune escape via PD-L1 in hepatocellular carcinoma**

Feng Qi^#^, Xiaojing Du^#^, Zhiying Zhao^#^, Ding Zhang, Mengli Huang, Yuezong Bai, Biwei Yang^*^, Wenxing Qin^*^, Jinglin Xia^*^

^*^Corresponding authors:

Prof. Jinglin Xia, [xiajinglin@fudan.edu.cn](mailto:xiajinglin@fudan.edu.cn);

Prof. Wenxing Qin, [qinwenxingqwx@163.com](mailto:qinwenxingqwx@163.com);

Prof. Biwei Yang, [yang.biwei@zs-hosptial.sh.cn](mailto:yang.biwei@zs-hosptial.sh.cn).

^#^Feng Qi, Xiaojing Du and Zhiying Zhao contributed equally to this research work.

**Supplementary Material includes:**

Supplementary Methods

Supplementary Figure S1 to Figure S6

Supplementary Table S1 to Table S2

**Supplementary Methods**

***Hematoxylin and eosin (HE) staining***

HE staining was performed according to the instructions of the staining kit (C0105; Beyotime Biotech, ).

**Supplementary Figures and Figure Legends**


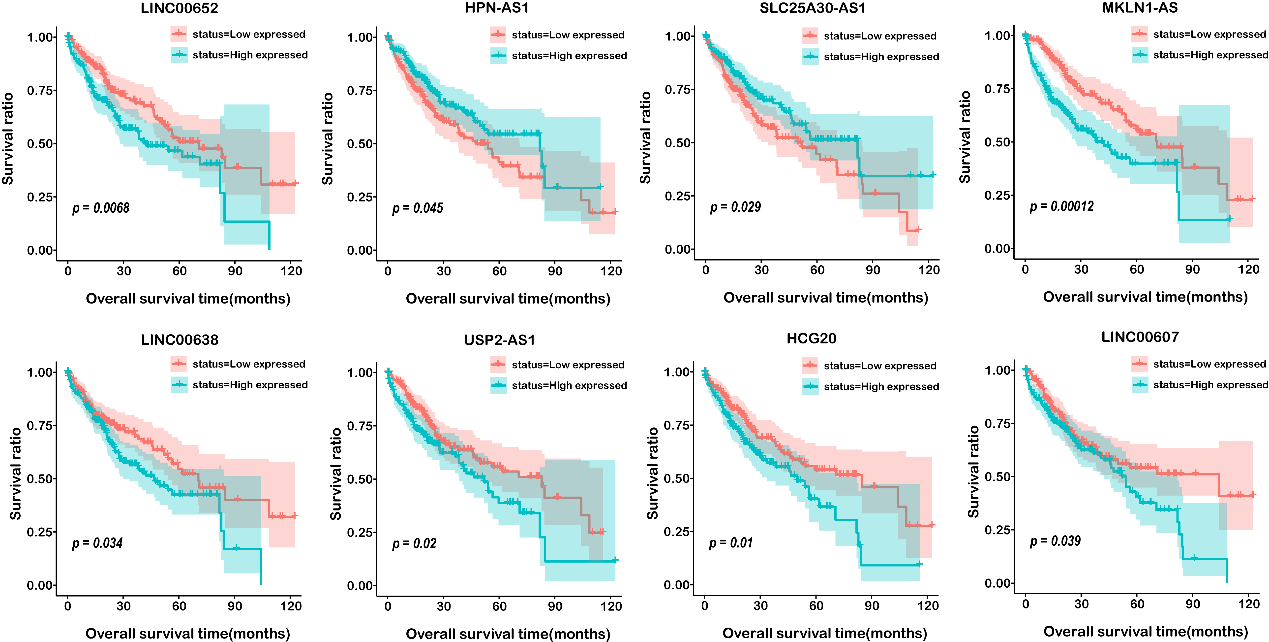


**Figure S1. The correlation between OS and hub lncRNAs in HCC.**


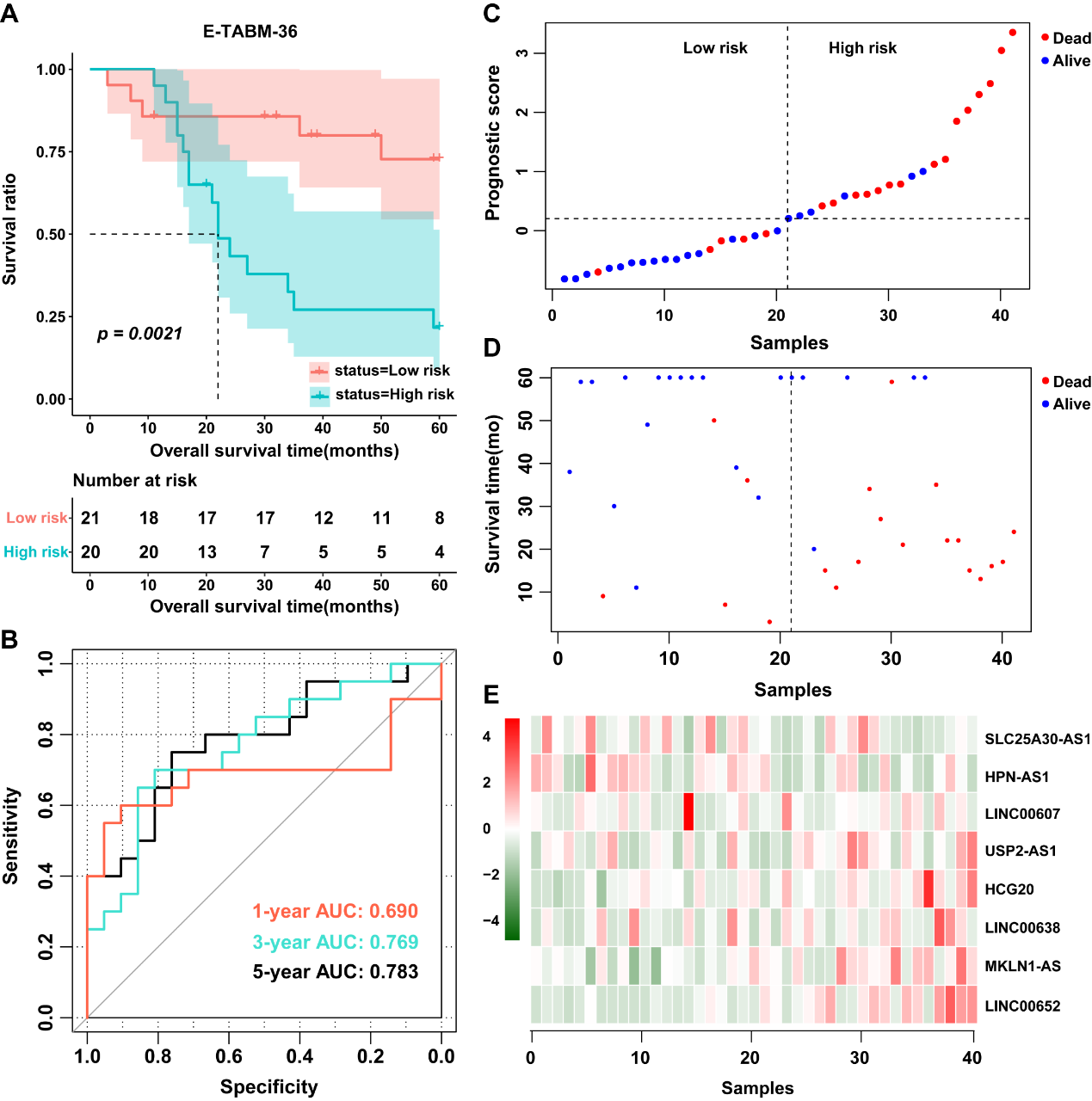


**Figure S2. Validation of the eight-lncRNAs PS model in E-TABM-36 cohort.** A. K-M curves result. B. The AUC of the PS for the prediction of 1, 3, 5-year survival rate of HCC. C&D. Risk survival status plot. E. The heatmap of the hub lncRNAs in validation cohort. PS, prognostic score; K-M, Kaplan-Meier; AUC, area under the curve; HCC, hepatocellular carcinoma.


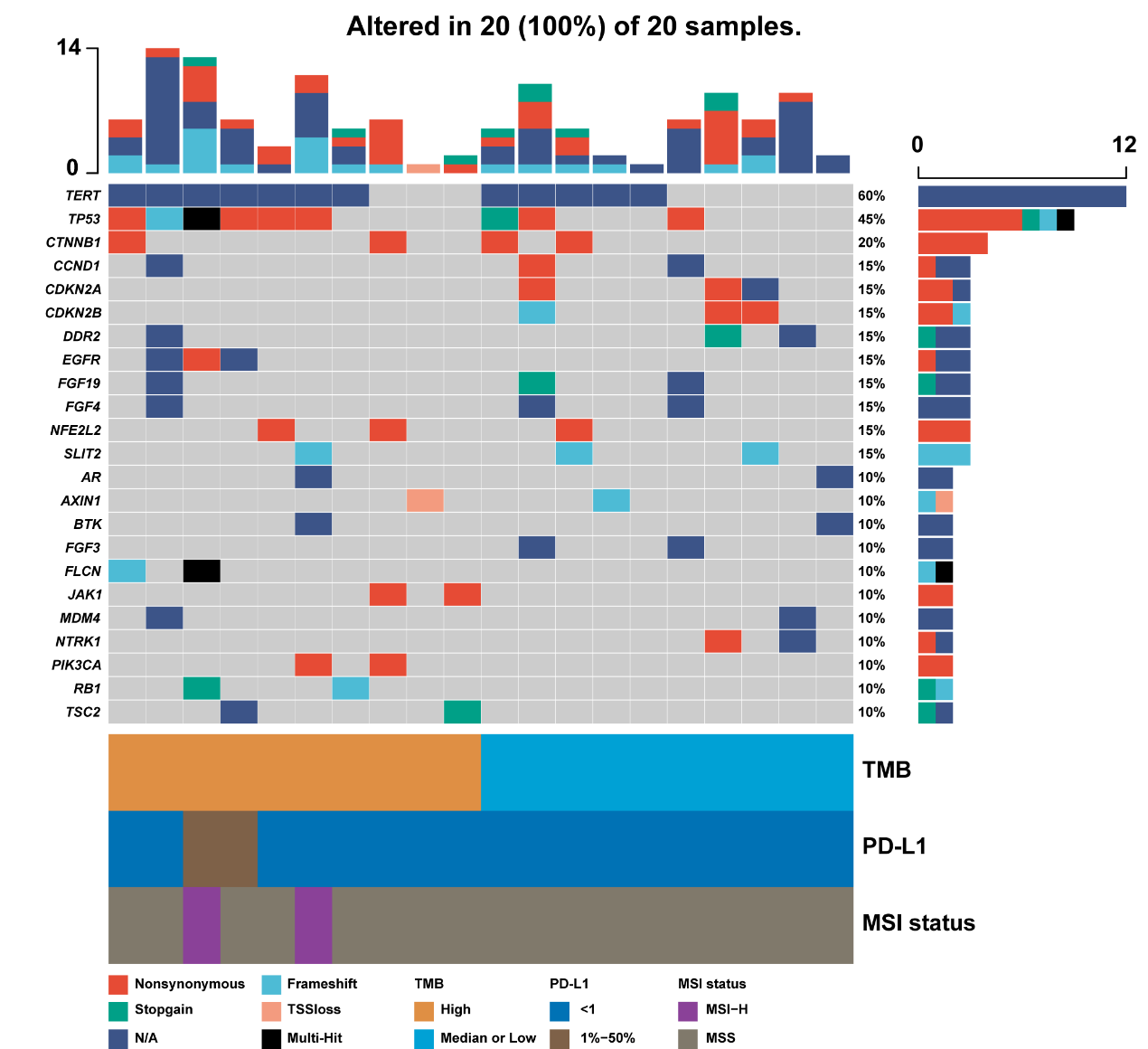


**Figure S3. NGS result of 20 patients with HCC.** MSI-H, microsatellite instability-high; MSS, microsatellite stability; NGS, second generation sequencing.

**
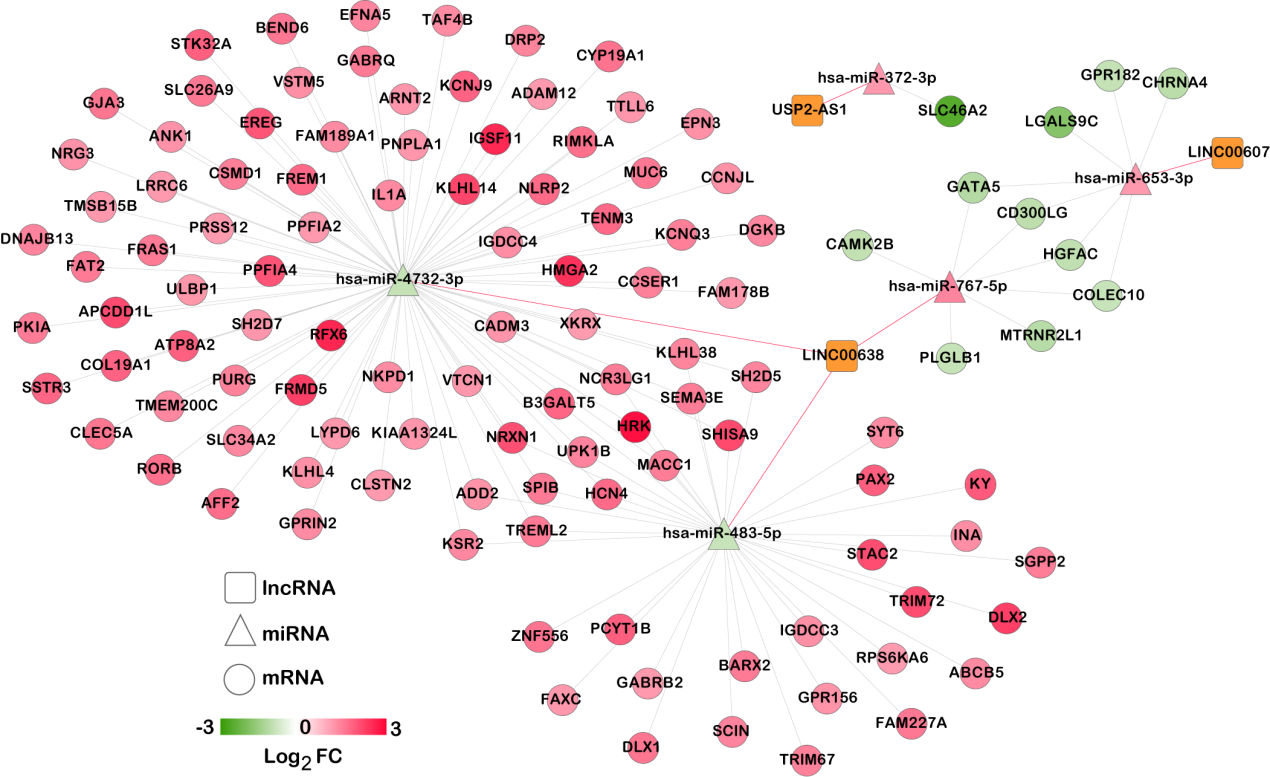
Figure S4. The ceRNA network visualized by Cytoscape.** Tetragonum, triangle and circular nodes denoted lncRNA, miRNA, and mRNA, respectively.


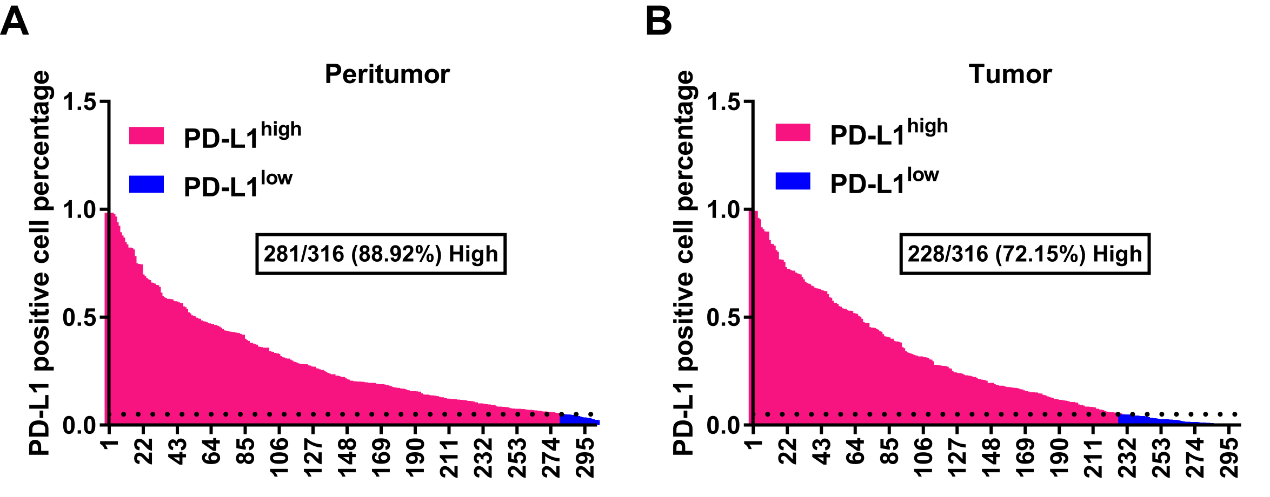


**Figure S5. PD-L1 expression in HCC. A.** The percentage of PD-L1^+^ cells in tumor tissues of HCC. **B.** The percentage of PD-L1^+^ cells in peritumor tissues of HCC. HCC, hepatocellular carcinoma.


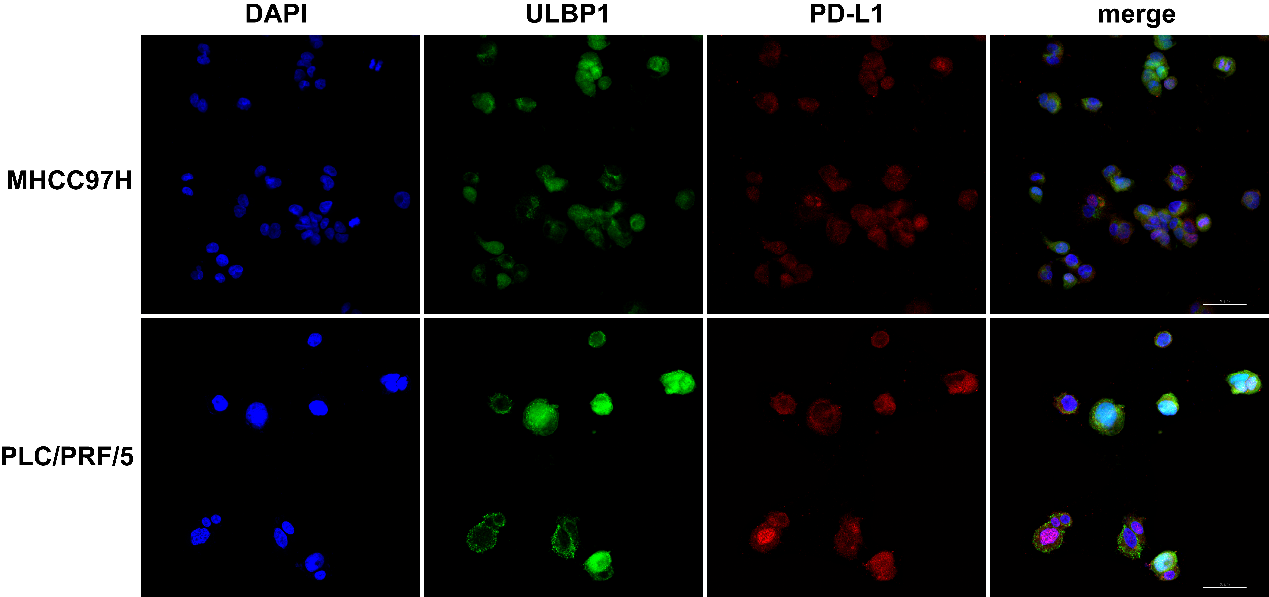


**Figure S6. Co-localization analysis of ULBP1 and PD-L1 in HCC cell lines.** ULBP1 and PD-L1 was co-expressed in MHCC97H and PLC/PRF/5 cells. HCC, hepatocellular carcinoma.

**
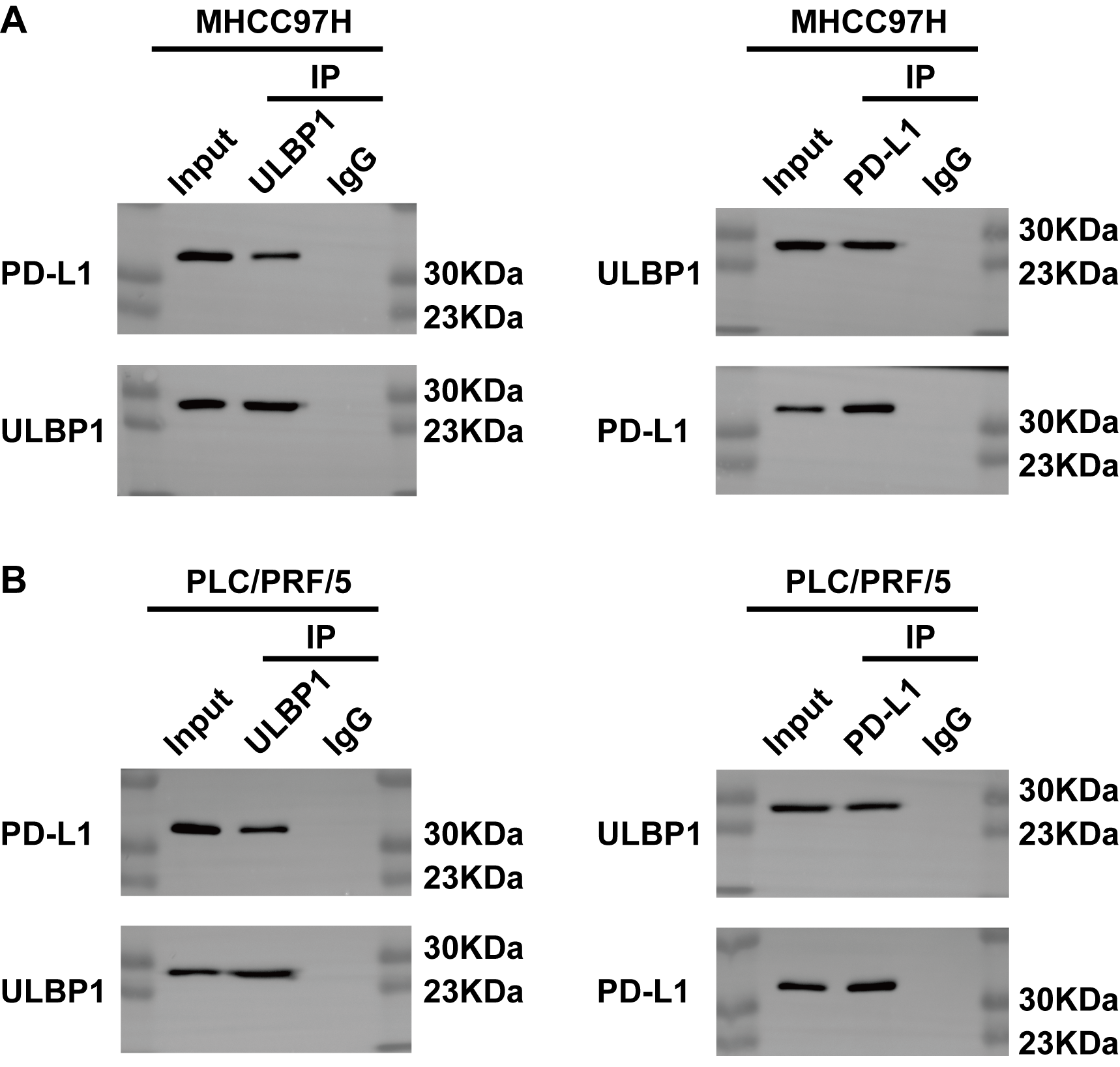
**

**Figure S7. Co-immunoprecipitation (Co-IP) of ULBP1 and PD-L1 in HCC cell lines.** The relationship between ULBP1 and PD-L1 was validated in MHCC97H (A) and PLC/PRF/5 cells (B). HCC, hepatocellular carcinoma, IgG was used as control.
